# The non-canonical ubiquitin activating enzyme UBA6 suppresses epithelial-mesenchymal transition of mammary epithelial cells

**DOI:** 10.18632/oncotarget.20900

**Published:** 2017-09-15

**Authors:** Xianpeng Liu, Limin Sun, Demirkan B. Gursel, Chonghui Cheng, Sui Huang, Alfred W. Rademaker, Seema A. Khan, Jun Yin, Hiroaki Kiyokawa

**Affiliations:** ^1^ Department of Pharmacology, Northwestern University, Chicago, Illinois 60611, USA; ^2^ Department of Pathology, Northwestern University, Chicago, Illinois 60611, USA; ^3^ Division of Hematology/Oncology, Northwestern University, Chicago, Illinois 60611, USA; ^4^ Department of Cell and Molecular Biology, Northwestern University, Chicago, Illinois 60611, USA; ^5^ Department of Preventive Medicine, Northwestern University, Chicago, Illinois 60611, USA; ^6^ Department of Surgery, Northwestern University, Chicago, Illinois 60611, USA; ^7^ Robert H. Lurie Comprehensive Cancer Center, Northwestern University, Chicago, Illinois 60611, USA; ^8^ Department of Chemistry, Center for Diagnostics and Therapeutics, Georgia State University, Atlanta, Georgia 30303, USA; ^9^ Current/Present address: Lester and Sue Smith Breast Center, Baylor College of Medicine, Houston, TX 77030, USA

**Keywords:** ubiquitination, breast cancer, EMT, Rho GTPase, cell cycle

## Abstract

Ubiquitination plays critical roles in the regulation of oncoproteins and tumor suppressors during carcinogenesis. The two ubiquitin activating enzymes (E1) in human genome, UBA1 and UBA6, initiate ubiquitination by ATP-dependent activation of ubiquitin. Recent evidence suggests that UBA1 and UBA6 play partially overlapped yet distinct roles in controlling the proteome. Here we demonstrate that ubiquitination pathways initiated specifically by UBA6 set a suppressive barrier against critical steps of mammary carcinogenesis such as loss of polarity, anoikis resistance and epithelial-mesenchymal transition (EMT). Mammary epithelial MCF-10A cells expressing shRNA against *UBA6* fail in establishing cell cycle arrest in response to detachment from extracellular matrix, confluency with fully engaged cell-cell contact or growth factor deprivation. Moreover, UBA6-deficient MCF-10A cells undergo spontaneous EMT under growth factor deprivation and exhibit accelerated kinetics of TGF-β-induced EMT. The Rho-GTPase CDC42 is one of the specific targets of UBA6-initiated ubiquitination and plays a key role in the function of UBA6 in controlling epithelial homeostasis, since a CDC42 inhibitor, ML141, rescues UBA6-deficient cells from the EMT phenotype. Immunohistochemical analysis of human breast cancer tissues demonstrates that 38% of invasive carcinomas express low or undetectable expression of UBA6, suggesting that downregulation of this non-canonical E1 plays a role in breast cancer development.

## INTRODUCTION

Ubiquitination is the process of covalent conjugation of ubiquitin (UB) to cellular proteins mediated by E1 (UB activating enzyme)-E2 (UB conjugating enzyme)-E3 (UB ligase) enzyme cascade [1, 2]. Ubiquitination plays critical roles in various diseases including cancer [3]. E1 catalyzes the formation of the thioester bond between the carboxyl terminus of UB and the active site cysteine of E1 in the presence of ATP [4, 5], initiating UB transfer to E2 and subsequently to target proteins that are recruited by E3. The mammalian genome contains two E1 genes, which encode the canonical E1 enzyme UBA1 and the non-canonical E1 enzyme UBA6 [4–6]. While both UBA1 and UBA6 are expressed ubiquitously, UBA1 expression in most cell types is several-fold higher than UBA6 expression [7]. Among ~40 E2 enzymes, UBE2Z/Use1 specifically interacts with UBA6, while some E2s are shared by the two E1s [8]. Previous studies identified several targets for UBA6-initiated ubiquitination, such as RGS4, RGS5, UBE3A/E6-AP and Shank3 [9, 10]. To better understand the biological roles of the dual E1 ubiquitination system, we recently conducted proteome-wide screens for target proteins of UBA1- and UBA6-initiated ubiquitination, using the Orthogonal UB Transfer (OUT) technology, and demonstrated partially overlapped yet distinct pools of substrates for UBA1 and UBA6 [11]. This work has provided direct evidence of non-redundant functions of the two E1s, which were suggested previously by biological data. For instance, rodent cells carrying a temperature-sensitive *Uba1* mutation exhibit rapid chromosomal instability, S/G2 cell cycle arrest and marked accumulation of normally short-lived proteins at non-permissive temperatures [12–15]. *Uba6* knockout mice are embryonic lethal and mice with brain-specific disruption of *Uba6* show defects in neuronal development and an autism-like phenotype [10, 16, 17]. UBA6 activates not only UB but also another UB-like modifier, FAT10 [17]. FAT10 conjugation and subsequent degradation of target proteins are involved in the control of immunity [17, 18]. Interestingly, *Fat10-*null mice are viable and exhibit only modest metabolic changes [19, 20], suggesting that UBA6-dependent ubiquitination, but not FAT10 conjugation, is essential for embryonic development.

The roles for the two E1s in epithelial biology and oncogenesis are largely unknown. It has been reported that *UBA1* silencing or pharmacologic inhibition of UBA1 causes cell death in myeloma and leukemia cell lines and primary leukemia cells, and delays tumor growth in SCID mice from leukemia xenografts [21]. UBA1 inhibition in HCT116 human colon cancer cells results in cell death and *UBA1* knockdown inhibits tumor growth from HCT116 xenografts [22]. Those data imply an oncogenic role of UBA1 at least in leukemia, myeloma and colon cancer [21–24]. Our OUT screens have revealed that pathways associated with UBA6-specific ubiquitination are linked significantly to cell morphogenesis, adhesion, motility, survival and stress responses [11]. Consistently, *UBA6* silencing in mammary epithelial MCF-10A cells results in impaired cell polarity and failed formation of lumen [11], suggesting a key role of UBA6 in mammary epithelial morphogenesis. During normal acinar morphogenesis, death of inner cells induced by the detachment from extra cellular matrix (ECM), i.e., anoikis, results in the hollow lumen formation [25, 26]. In contrast, anoikis resistance in precancerous cells leads to anchorage-independent cell survival, filling of glandular structure, and eventually the development of invasive cancer [27–29]. In the present study, we demonstrate that under growth factor deprivation, UBA6-deficient cells exhibit continuous cell cycle progression and spontaneous EMT with concomitant stabilization of UBA6-specific ubiquitination targets such as ezrin, CDC42 and CUGBP1. These observations suggest that UBA6-initiated ubiquitination normally prevents mammary epithelial cells from undergoing deregulated proliferation and EMT. Moreover, our tissue microarray analysis shows that the expression of UBA6 is low or undetectable in a substantial population of invasive breast cancer tissues, suggesting the cancer-associated roles for the non-canonical E1.

## RESULTS

### UBA6 is required for the mammary epithelial morphogenesis

**Figure 1:**
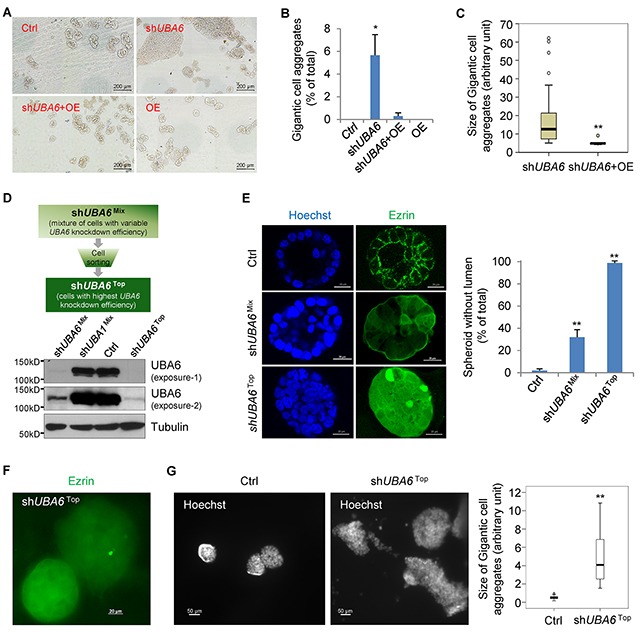
*UBA6* knockdown perturbs acinar morphogenesis and leads to formation of gigantic cell aggregates in mammary epithelial 3-D culture **(A)** Representative pictures that are acquired by TissueFAXS 200 with 10x magnification for the measurement of the size of acini and gigantic cell aggregates. **(B)** Percentages of gigantic cell aggregates (>0.04 mm^2^ in size of area) in total acini analyzed from (A). **(C)** Quantification of the size of gigantic cell aggregates. The images were acquired by TissueFAXS 200 from three independent experiments. The size of gigantic cell aggregates was quantified using imageJ software. Box-and-whisker plots show significant difference in the distribution of size in two groups (Mann–Whitney U = 25, n1 = 70, n2 = 5, P < 0.01). **(D)** Flow cytometric sorting for MCF10A sh*UBA6*^Top^ cells, which showed most efficient knockdown of UBA6. **(E)** Representative pictures of Immunofluorescence microscopy (10x magnification) for ezrin and chromosomal DNA (via Hoechst 33342) in spheroids formed by MCF10A control cells, sh*UBA6*^Mix^ cells and sh*UBA6*^Top^ cells in 3D culture at day 14 showing complete loss of lumen formation in sh*UBA6*^Top^ cells. **(F)** Immunofluorescence microscopy (10x magnification) for ezrin in gigantic cell aggregates formed by sh*UBA6*^Top^ cells in 3-D culture at day 14. **(G)** Gigantic cell aggregates shown by immunofluorescence microscopy for chromosomal DNA in 3-D cultures of MCF10A control cells and sh*UBA6*^Top^ cells at day 12. The sizes of gigantic cell aggregates were quantified using ImageJ software. Box-and-whisker plots show significant difference in the distribution of size in two groups (Mann–Whitney U test, two tailed. n=30 and 16 for Ctrl and sh*UBA6*^Top^, respectively. ^**^P < 0.001). In (B) and (E), error bars show SEM of at least three independent experiments and asterisks indicate statistical significance (p<0.05).

The method of culturing mammary epithelial cells in 3-D with Matrigel has been widely used to recapitulate glandular morphogenesis *in vitro* [25, 26]. We previously used the system to show that *UBA6* silencing in human mammary epithelial MCF-10A cells inhibits lumen formation [11]. These data suggested an indispensable role of UBA6 in mammary epithelial morphogenesis and prompted us to further characterize the effects of UBA6-deficiency on epithelial cell regulation. After 14-day culture in 3-D, parental MCF10A cells formed typical acini or ductal-like spheroids (Figure 1A, Ctrl). MCF-10A cells stably expressing anti-*UBA6* shRNA formed similar structures, but also developed a number (~5%) of tumor-like gigantic aggregates (sh*UBA6*, Figure 1A and 1B). In addition, approximately 30% of acini formed in the sh*UBA6* culture did not exhibit hollow lumen (shown as sh*UBA6*^Mix^ in Figure 1E), as we reported previously [11]. Forced expression of UBA6 cDNA in cells expressing anti-*UBA6* shRNA not only restored the formation of hollow lumen but also abrogated the formation of gigantic cell aggregates (Figure 1A–1C). These data suggest that the morphological impact of sh*UBA6* was indeed due to UBA6 deficiency, rather than off-target effects of the shRNA. The drug-selected cell population after lentiviral transduction of sh*UBA6* is a mixture of cells with variable efficiency of *UBA6* knockdown. Since only a fraction of these cells form gigantic cell aggregates, we postulated that cells with most efficient *UBA6* knockdown accounted for the formation of tumor-like cell aggregates. Thus, we performed flow cytometry for expression of green fluorescent protein (GFP) from the GIPZ vector, and sorted a population of cells with highest GFP intensity, which indeed exhibited more effective *UBA6* knockdown than the original drug-selected population (Figure 1D). We nominated the sorted cell population as sh*UBA6*^Top^ and the original drug-selected cell population as sh*UBA6*^Mix^, thereafter. Essentially all spheroids formed in 3-D culture of sh*UBA6*^Top^ cells failed in lumen formation and showed marked cytoplasmic accumulation of ezrin, in contrast to enriched localization of ezrin to plasma membranes of control cells (Figure 1E). Most spheroids of sh*UBA6*^Top^ cells became huge cell aggregates within 21 days of 3-D culture (Figure 1F and 1G). These data not only confirmed the role for UBA6 in acinar morphogenesis but also suggested that UBA6 deficiency promoted deregulated proliferation of epithelial cells in the Matrigel-based 3-D environment.

### UBA6 depletion suppresses contact inhibition of the cell cycle

To further characterize tumor-like aggregate formation induced by *UBA6* silencing, we isolated acini with normal appearance or gigantic cell aggregates from 3-D cultures, prepared cell suspensions from them, and incubated in conventional monolayer culture (Supplementary Figure 1A). Cells isolated from normal acini in control culture displayed the typical cobblestone-like epithelial monolayer, so did cells from normally formed acini in sh*UBA6* cultures (Supplementary Figure 1A, 1^st^ and 2^nd^ rows). In contrast, when cells from gigantic tumor-like aggregates were grown to high density, we observed numerous “islands” of cell clusters surrounded by typical epithelial monolayer (Supplementary Figure 1A, 3^rd^ row). Moreover, cells in the clusters continued proliferation and stacked up in piles with active incorporation of BrdU into their replicating DNA (Supplementary Figure 1A, 2^nd^ column). In contrast, cells that formed typical monolayer in confluency showed minimal BrdU incorporation. Cells within the clusters exhibited lower expression of the epithelial marker E-cadherin and higher expression of the mesenchymal marker Vimentin (Supplementary Figure 1B). These data suggest that UBA6 depletion promotes epithelial-mesenchymal transition (EMT), which could play a role in perturbed acinar morphogenesis of UBA6-deficient MCF-10A cells in 3-D culture. We also examined sh*UBA6*^Top^ cells in monolayer culture and found that those cells continued to proliferate without contact inhibition. Cell cycle regulators including CDK1, CDK4 and phosphorylated Rb were upregulated in sh*UBA6*^Top^ cells, compared with control cells and sh*UBA6*^Mix^ cells (Supplementary Figure 2A). Furthermore, the morphology of sh*UBA6*^Top^ cells in monolayer culture was quite distinct from that of typical epithelial cells, displaying a spindle-like mesenchymal morphology (Supplementary Figure 2B). The mesenchymal characteristics of sh*UBA6*^Top^ cells were further confirmed by upregulation of N-cadherin and vimentin and downregulation of E-cadherin and γ-catenin (Supplementary Figure 2C). Furthermore, sh*UBA6*^Top^ cells exhibited more profound motility in a wound-healing assay (Supplementary Figure 2D). According to these data, we hypothesized that complete depletion of UBA6 induced spontaneous EMT in MCF-10A cells.

### Effects of UBA6 depletion under growth factor deprivation

**Figure 2:**
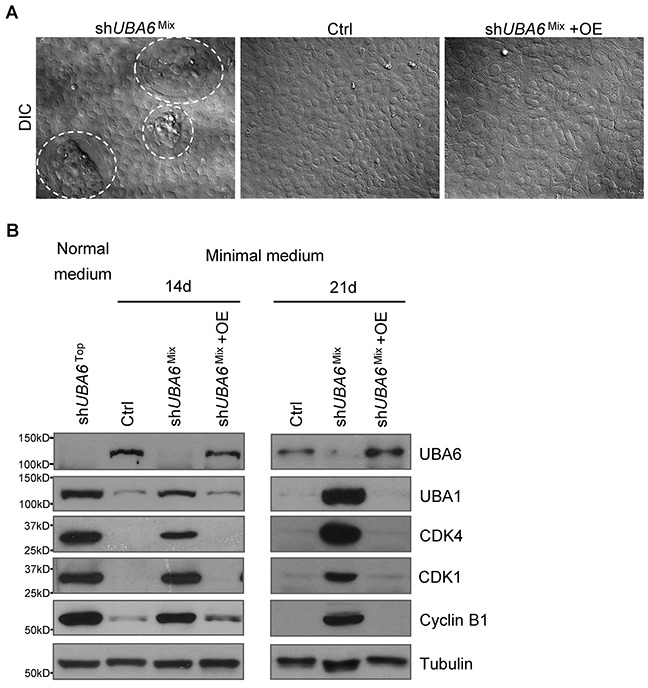
sh*UBA6*^Mix^ cells in minimal medium spontaneously form “island”-like clusters with upregulation of cell cycle regulators **(A)** Representative pictures of the formation of “island”-like cell clusters (circulated with dashed white lines) in confluent sh*UBA6*^Mix^ cells that were cultured in minimal medium for 14 days. See Materials and Methods for detailed information about the medium and cell lines. **(B)** MCF10A control cells, sh*UBA6*^Mix^ cells and sh*UBA6*^Mix^ cells with UBA6 cDNA overexpression (OE) were cultured in minimal medium for 14 or 21 days, followed by immunoblotting detection of the indicated proteins. The proteins from sh*UBA6*^Top^ cells that were cultured in normal medium were used as control for comparison.

While sh*UBA6*^Mix^ cells showed impaired acinar morphogenesis in 3-D culture (Figure 1A and 1E), those cells in monolayer culture did not show any appreciable change in morphology or proliferation (Supplementary Figure 1A and 2B). We speculated that impact of incomplete UBA6 depletion varied depending on cellular context, especially the extracellular environment. During spheroidal growth in 3-D culture, detachment of the inner cells from ECM leads to the lack of growth factors, nutrition starvation, and hypoxia. These microenvironmental changes cause various stresses, consequently leading to anoikis and formation of the hollow lumen characteristic of glandular morphogenesis [25–29]. On the other hand, MCF10A cells in monolayer culture are exposed to relative hyperoxia and excess growth factors and nutrients directly from the complete culture medium supplemented with horse serum plus epidermal growth factor (EGF), insulin, hydrocortisone, and cholera toxin. Thus, we next examined MCF-10A cells cultured in medium without supplementation of EGF, insulin, hydrocortisone and cholera toxin (called “minimal medium” thereafter). This condition of growth factor deprivation is known to induce cell cycle arrest in G_1_ [30], enhance anoikis of detached cells [31, 32], and alter epithelial cell morphology [33]. In monolayer culture with the complete medium, sh*UBA6*^Mix^ cells proliferated in a manner similar to control MCF-10A cells. After switching to minimal medium, control MCF10A cells showed growth arrest in 3 days (Figure 2A). However, sh*UBA6*^Mix^ cells started forming the “island”-like clusters within 3-5 days of culture in minimal medium (Figure 2A). These clusters were morphologically similar with those formed by sh*UBA6* cells that were isolated from gigantic cell aggregates in 3-D culture and incubated in the complete medium (Supplementary Figure 1A). We did not observe such changes in either control cells or sh*UBA6*^Mix^ cells with forced expression of UBA6 cDNA (+OE). These cell clusters continued expanding and eventually almost all sh*UBA6*^Mix^ cells exhibited sh*UBA6*^Top^-like mesenchymal morphology after 21 days of culture in minimal medium (Supplementary Figure 3A). This reflects continuous proliferation after completion of EMT, as the sh*UBA6*^Mix^ cells within the clusters demonstrated strong staining of proliferation marker Ki67 (Supplementary Figure 3B). Moreover, sh*UBA6*^Mix^ cells in minimal medium showed marked increases in essentially all cell cycle-regulatory proteins examined, such as D-, E- and B-type cyclins, CDK1, CDK2, CDK4, CDC25A and CDC25C, as well as RB phosphorylation (Figure 2B and Supplementary Figure 4). These data suggest UBA6 depletion causes ectopic cell cycle progression under growth factor deprivation.

### UBA6 depletion promotes EMT in cooperation with growth factor deprivation or TGF-β

**Figure 3:**
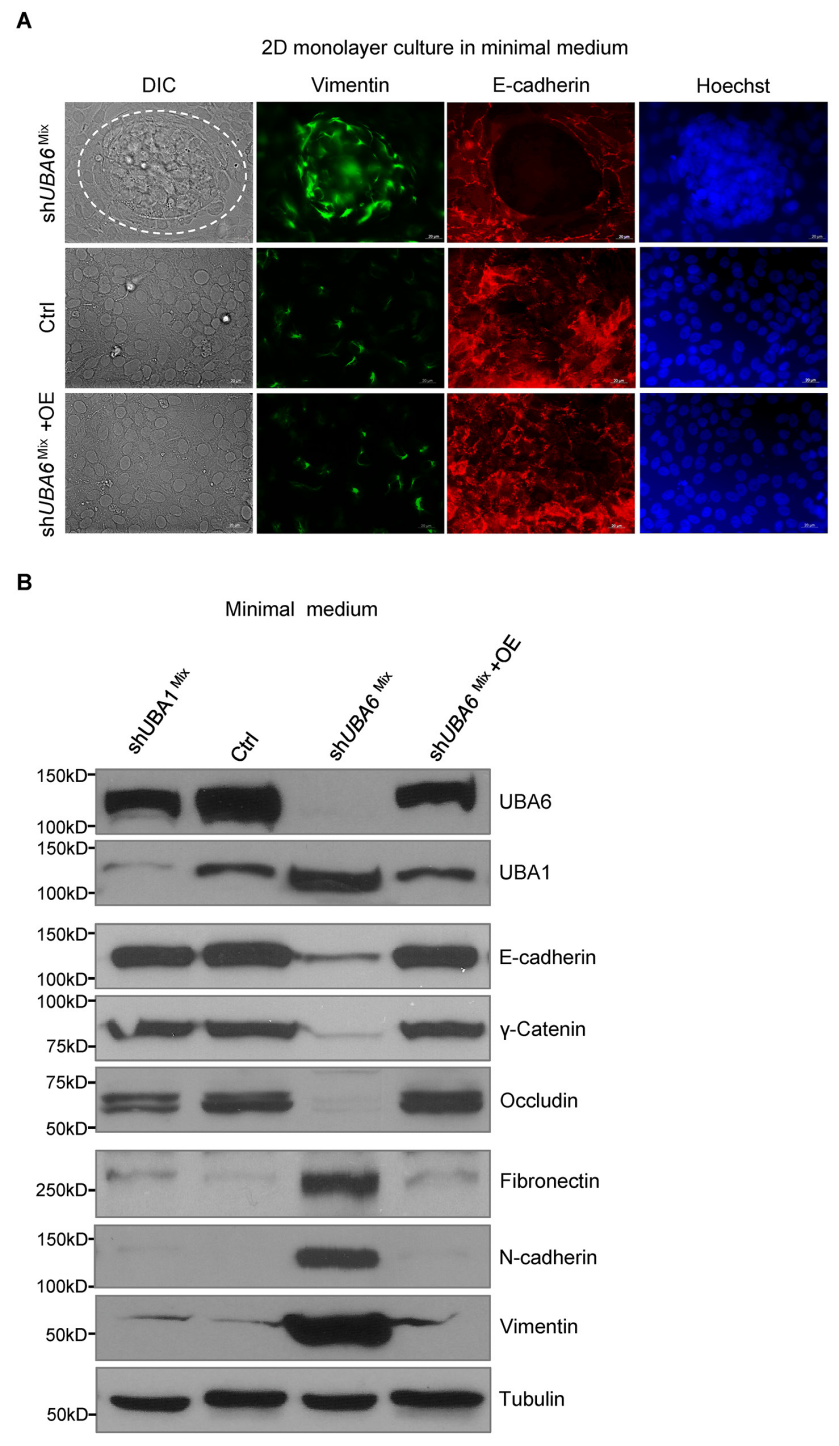
Growth factor deprivation induces EMT in sh*UBA6*^Mix^ MCF10A cells **(A)** Immunofluorescence microscopy for E-cadherin, vimentin and chromosomal DNA (via Hoechst 33342) in MCF10A control cells, sh*UBA6*^Mix^ cells and sh*UBA6*^Mix^ +OE cells that were cultured in minimal medium for 14 days. **(B)** Immunoblotting detection of EMT markers in MCF10A control cells, sh*UBA1*^Mix^ cells, sh*UBA6*^Mix^ cells and sh*UBA6*^Mix^ +OE cells that were cultured in minimal medium for 14 days.

After prolonged culture in minimal medium, sh*UBA6*^Mix^ cells displayed a mesenchymal morphology, which appeared identical to that of sh*UBA6*^Top^ cells. We next examined whether sh*UBA6*^Mix^ cells within the clusters were indeed undergoing EMT in response to growth factor deprivation. Immunofluorescence microscopy demonstrated downregulation of the epithelial marker E-cadherin and upregulation of the mesenchymal marker Vimentin within the clusters of sh*UBA6*^Mix^ cells after switching to minimal medium (Figure 3A). Consistently, immunoblotting demonstrated significant decreases in levels of the epithelial markers (E-cadherin, γ-Catenin and Occludin) and increases in levels of the mesenchymal markers (Fibronectin, N-cadherin and Vimentin) in sh*UBA6*^Mix^ cells (Figure 3B). These effects of sh*UBA6* on EMT were abolished by the forced expression of *UBA6* cDNA in sh*UBA6*^Mix^ cells (Figure 3B), verifying that the observed EMT induction was not a consequent of off-target effects. In contrast, *UBA1* silencing had minimal effects on the EMT markers, as shown in sh*UBA1*^Mix^ cells.

**Figure 4:**
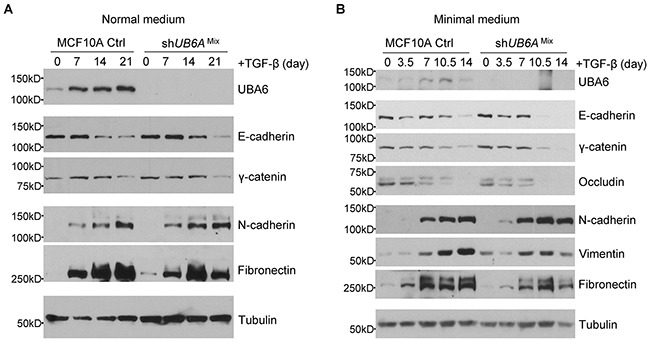
TGF-beta induced EMT is accelerated in sh*UBA6*^Mix^ MCF10A cells in minimal medium The MCF10A control (Ctrl) cells and sh*UBA6*^Mix^ cells were cultured in normal medium (**A**) or in minimal medium for 14 days (**B**). The EMT was induced by adding 1ng/mL TGF-β to normal or minimal medium. The cells were collected at the indicated time points for by immunoblotting detection of EMT markers.

TGF-β has been extensively studied as a primary inducer of EMT [34]. We thus examined the effects of UBA6 silencing on TGF-β-induced EMT in the presence or absence of growth factors. After TGF-β treatment in normal medium, no appreciable difference between control cells and sh*UBA6*^Mix^ cells was observed regarding the levels of EMT markers. However, we observed accelerated kinetics of the decreases in epithelial markers and the increases in mesenchymal markers in shUBA6^Mix^ cells treated with TGF-β in minimal medium (Figure 4). These data further confirm the role for UBA6 in the regulatory process of EMT.

### CDC42 is a substrate of UBA6-initiated ubiquitination

**Figure 5:**
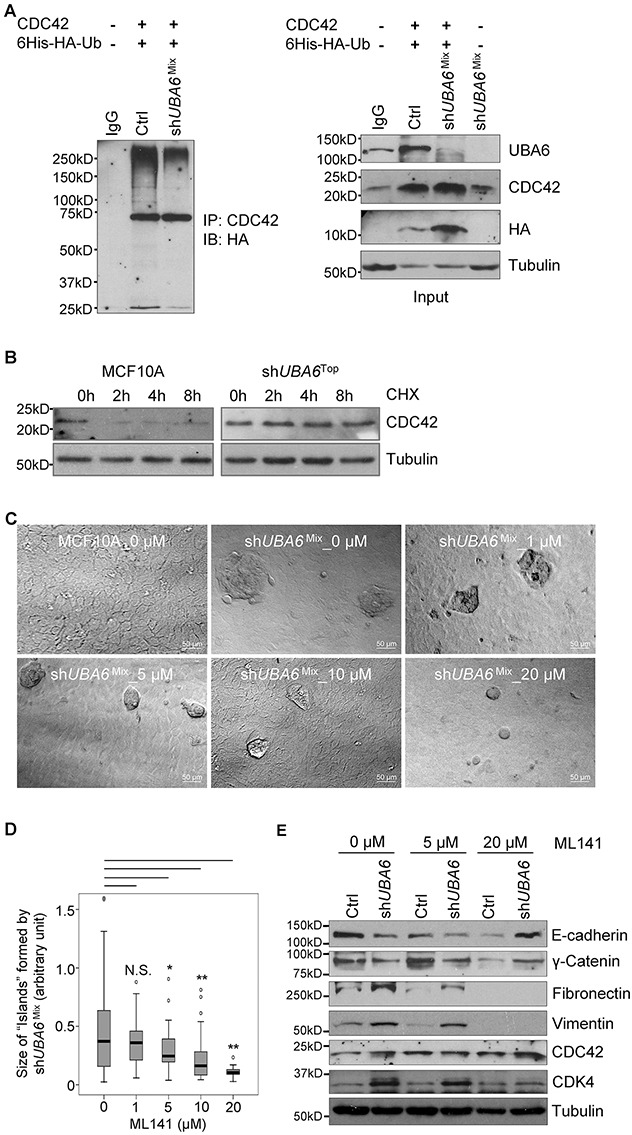
The Rho-GTPase CDC42 is a substrate of UBA6-dependent ubiquitination and is involved in EMT induced by UBA6 deficiency and growth factor deprivation **(A)** UBA6 depletion downregulates polyubiquitinated forms of CDC42. HEK293 cells were transfected with plasmids for expression of CDC42 and poly-histidine-HA-tagged ubiquitin (Ub) and treated with 10 μM MG132 for 90 min. Cells were then harvested and analyzed by immunoprecipitation (IP) with anti-CDC42 antibody, followed by immunoblotting (IB) for the HA tag. IgG, control immunoprecipitation with normal rabbit IgG. The right panels indicate immunoblotting with total cell lysates for the indicated proteins. **(B)** Uba6 targets CDC42 to degradation. MCF10A control cells and sh*UBA6*^Top^ cells were treated with 100 μg/mL cycloheximide for the indicated hours, and then examined by immunoblotting for CDC42 to determine its stability. **(C)** Formation of island-like cell clusters on monolayer of MCF10A-sh*UBA6*^Mix^ cells after 7-day incubation in minimal medium supplemented with the CDC42 inhibitor ML141 at the indicated doses. **(D)** Box-and-whisker plots showing significant differences in sizes of island-like clusters after ML141 treatment (n1 = 107, n2 = 63, n3=48, n4= 61, n5=26). ^*^p<0.05; ^**^p<0.01; N.S., not significant. **(E)** ML141-mediatd suppression of the EMT process in sh*UBA6*^Mix^ cells induced by incubation in minimal medium. Cells were harvested after 11-day incubation with ML141 at indicated doses in minimal medium, and analyzed by immunoblotting.

The Rho family GTPases such as CDC42 and RAC1 are involved in the regulation of epithelial cell polarity, via their functional interaction with the Par3-Par6-aPKC polarity complex [35]. EMT reprograms the function of the polarity complex from maintenance of epithelial cell shape to establishment of invasive cell phenotype, in which CDC42 and RAC1 play rate-limiting roles [36]. Our OUT-based screens identified CDC42 as a candidate of UBA6-specific ubiquitination targets [11]. Therefore, we hypothesized that the lack of UBA6-initiated CDC42 ubiquitination was involved in the EMT phenotype of growth factor-deprived sh*UBA6*^Mix^ cells. Indeed, CDC42 protein was upregulated significantly in sh*UBA6*^Mix^ cells cultured in minimal medium (Supplementary Figure 5), which was consistent with the hypothesis. To confirm that CDC42 was ubiquitinated in a UBA6-dependent manner, we expressed CDC42 and HA-tagged ubiquitin in control and sh*UBA6* HEK293 cells by plasmid transfection. CDC42 immunoprecipitated from control cells exhibited robust polyubiquitinated forms of CDC42 (Figure 5A). In contrast, CDC42 immunoprecipitates from sh*UBA6* cells showed significantly decreased polyubiquitinated forms, verifying that polyubiquitination of CDC42 is dependent on UBA6. We also observed a significant increase in the stability of CDC42 protein in sh*UBA6*^Top^ cells (Figure 5B), suggesting that UBA6-dependent ubiquitination targets CDC42 to degradation. We then assessed the involvement of CDC42 in EMT of UBA6-deficient cells under growth factor deprivation. Incubation of sh*UBA6*^Mix^ cells with the CDC42 inhibitor ML141 in minimal medium suppressed island-like cluster formation in a dose-dependent manner (Figure 5C and 5D). Suppression of EMT by ML141 was confirmed by immunoblotting for the epithelial and mesenchymal markers (Figure 5E). These data suggest that UBA6-initiated ubiquitination normally downregulates CDC42 by proteasomal degradation, and that upregulation of CDC42 in UBA6-deficient cells plays a role in facilitating EMT.

### Downregulation of UBA6 in invasive breast cancer

**Figure 6:**
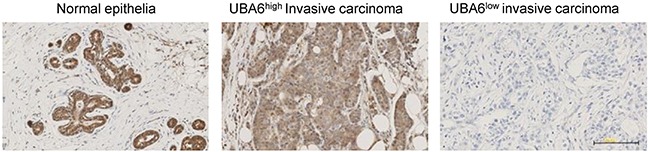
UBA6 is downregulated in a subset of invasive breast cancers Representative images of immunohistochemical detection of UBA6 protein in normal human breast epithelial tissues (n=14) and in invasive breast cancer tissues on tissue microarray (n=250).

Our data from MCF-10A cells suggest that perturbed UBA6 function may facilitate the development of invasive breast cancer. To evaluate this possibility and assess clinical relevance of the cellular phenotype of UBA6 deficiency, we examined the expression of UBA6 protein in normal mammary glands and invasive mammary carcinomas by conducting analysis of tissue microarrays (TMAs). First, we validated two UBA6 commercially available antibodies (1C11 from Abnova and AP16886b from Abgent), conducting immunohistochemistry (IHC) with paraffin embedded sections of HEK293 cells with stable overexpression of UBA6, HEK293 cells with stable expression of anti-UBA6 shRNA, and human testis. Testicular expression of UBA6 is known to have a distinct differentiation-associated pattern [37]. We then analyzed the expression of UBA6 in human mammary tissues with or without malignant changes. Luminal epithelial cells showed robust expression of UBA6 protein in all normal mammary tissues examined (n=14) (Figure 6). In contrast, UBA6 expression was much weaker or undetectable in 38% of total invasive breast cancer tissues examined (n=250). The downregulation of UBA6 was more significantly manifested in ER negative, PR negative, HER2 positive or triple negative breast cancers (Table 1). Taken together, these data are consistent with the hypothesis that UBA6 downregulation promotes cancer progression such as EMT and invasion in a population of human breast cancers.

**Table 1:** UBA6 expression in human invasive mammary carcinomas

Characteristics	All tumors	UBA6-high	UBA6-low	*P* value
	251	156 (62%)	95 (38%)	
ER				
Positive	237	155 (65%)	82 (35%)	
Negative	14	1 (7%)	13 (93%)	<0.0001
PR				
Positive	207	140 (68%)	67 (32%)	
Negative	44	16 (36%)	28 (64%)	0.0001
HER2				
Positive	25	6 (24%)	19 (76%)	0.0001
Negative	183	119 (65%)	64 (35%)	
Triple Negative				
Yes	10	1 (10%)	9 (90%)	0.0007
No	237	155 (65%)	82 (35%)	

## DISCUSSION

While all ubiquitination events in the cell are initiated either by UBA1- or UBA6-dependent UB activation, only limited information was available about whether and how these two E1s play distinct roles in the regulation of cell fate, e.g., developmental differentiation and oncogenic transformation. Here we demonstrate that UBA6 plays unique roles in suppressing EMT, a critical step of cancer progression toward invasion and metastasis. UBA6 depletion allows MCF-10A cells to continue cell cycle progression despite the loss of ECM support in 3-D culture, fully engaged cell-cell contact or deprivation of growth factors in monolayer culture. Deregulated cell cycle progression of UBA6-deficient cells is accompanied by phenotypic changes characteristic of EMT, i.e., mesenchymal-like morphology, downregulation of epithelial proteins (E-cadherin, γ-Catenin and Occludin) and upregulation of mesenchymal proteins (Fibronectin, N-cadherin and Vimentin). These profound effects of UBA6 deficiency suggest that UBA6 activity controls diverse signaling pathways involved in homeostatic coordination of polarity, proliferation and survival in response to the context of ECM and cell-cell adhesion.

Collectively, the phenotypes of UBA6-deficient mammary epithelial cells are analogous with those of anoikis-resistant invasive cancer cells. Anoikis plays a physiological role in hollow lumen formation during epithelial acinar morphogenesis. Anoikis resistance enables cancerous cells to survive and proliferate independently of ECM and leads to the invasive phenotype [27–29]. We found that UBA6 deficiency impairs lumen formation in MCF-10A 3-D cultures and results in formation of tumor-like cell aggregates, which are reminiscent of the development of invasive carcinomas at early stages. Carcinoma cells may achieve anoikis resistance by undergoing EMT [27–29]. EMT enables transformed cells to survive in an anchorage-independent manner and attain their ability of higher cell motility and invasion, leading to invasive and ultimately metastatic behaviors [27–29]. Our OUT screens identified a number of UBA6-specific ubiquitination targets that fall into several EMT-associated pathways [36, 38] such as “Cdc42 Signaling”, “RhoA Signaling”, and “Epithelial Adherens Junction Signaling”, and suggested that UBA6-dependent ubiquitination is critical for EMT [11]. Consistently, *UBA6* silencing promotes the EMT process in microenvironment-dependent manners (Supplementary Figures 1–4). We previously reported that UBA6-dependent polyubiquitination regulates the stability of ezrin [11], which plays a role in maintaining apico-basal polarity to ensure proper lumen formation during acinar morphogenesis [39, 40]. In the present study we have verified CDC42 as another UBA6-specific polyubiquitination substrate (Figure 5). CDC42 is known to be a major regulator of epithelial cell polarity, motility and EMT [36]. We demonstrate that CDC42 is stabilized and upregulated in UBA6-deficient cells (Figure 5B and Supplementary Figure 5). Our observation that the CDC42 inhibitor ML141 suppresses island-like cluster formation and EMT in growth factor-deprived sh*UBA6*^Mix^ cells indicates that polyubiquitination of this Rho-GTPase mediates significant part of UBA6-dependent control of epithelial cell homeostasis. Furthermore, we previously reported that the RNA binding protein CUGBP1/CELF1 (CUG RNA-binding protein and embryonically lethal abnormal vision-type RNA-binding protein 3-like factor 1) is a UBA6-specific ubiquitination target and CUGBP1 undergoes proteasomal degradation in response to polyubiquitination [11]. A recent study demonstrated that CUGBP1 is a central regulator of posttranscriptional programs that drive the EMT process [41]. Indeed, the study showed that CUGBP1 protein, but not mRNA is overexpressed in human breast cancer tissues, indicating a possibility that ubiquitin-dependent degradation of CUGBP1 is hampered in cancer cells. We observed that sh*UBA6*^Mix^ cells exhibited high levels of CUGBP1 protein upon prolonged growth factor withdrawal or TGF-β treatment (Supplementary Figure 6), suggesting the involvement of CUGBP1 in the progression of EMT. Taken together, these data suggest that UBA6-dependent ubiquitination forms a previously undefined mechanism of EMT suppression, controlling EMT-related downstream targets including Ezrin, CDC42 and CUGBP1.

ECM detachment in 3-D culture, as well as growth factor deprivation in monolayer culture, can trigger cell cycle arrest at G_1_ [27, 30]. Cells with UBA6 depletion exhibit upregulation of CDKs and cyclins even under growth factor deprivation (Figure 2B, Supplementary Figure 2A and Supplementary Figure 4), suggesting that UBA6 is required for context-dependent control of the cell cycle. It has been appreciated that cellular levels of cyclins and CDKs are controlled precisely by coordination of transcription/translation and ubiquitin-dependent degradation. While the present study suggests a nonredundant role of UBA6-dependent ubiquitination in cell cycle regulation, mechanisms of such control remains to be determined. It is possible that continued proliferation of UBA6-deficient MCF-10A cells under growth factor deprivation is dependent on completion of the spontaneous EMT process and observed changes in the cell cycle regulators simply reflects active proliferation rather than being a direct consequence of impaired UBA6-dependent ubiquitination. Our data also suggest a crosstalk between UBA6 and UBA1. We observed modest but significant increase of UBA1 protein in shUBA6 ^Mix^ cells in minimal medium (Figure 2, Figure 3B, Supplementary Figure 2A and Supplementary Figure 4), implying functional compensation by UBA1 in UBA6-deficient cells. It was reported that UBA1 stability is controlled by conjugation with FAT10, which is mediated by UBA6 and UBE2Z/Use1 [42]. Thus, further investigations are needed to determine how UBA1-initiated ubiquitination events contribute to the phenotypes of UBA6-deficient cells under various cellular contexts. Also, it is unknown which E2s cooperate with UBA6 in regulating EMT. Several E2s can accept UB from UBA6, including UBE2Z, UBE2D2 and BIRC6 [8, 11]. A recent report showed that UBE2Z is frequently overexpressed in lung cancer, proposing a cell cycle promoting ability of the E2 enzyme [43]. Another open question is whether the UBA6-UBE2Z-FAT10 axis plays a substantial role in the regulation of EMT and cell cycle progression.

The present study suggests that UBA6-dependent ubiquitination of cellular proteins mediates the coordination of epithelial cell homeostasis with extracellular signals and prevents ectopic survival and outgrowth in cytostatic microenvironments. Thus, our observations that a substantial fraction of human breast cancer tissues express low or undetectable levels of UBA6 protein suggest that UBA6 downregulation during carcinogenesis plays a previously undefined role in developing invasive phenotypes of breast cancer. While a couple of UBA6-dependent ubiquitination substrates, i.e., CDC42, ezrin and CUGBP1, are upregulated in cells with UBA6 depletion, it remains to be determined how downregulation of UBA6 activity impact the proteome of mammary epithelial cells during breast cancer development. Our OUT screens identified hundreds of potential substrates specific for UBA6-dependent ubiquitination, which will serve as a basis for elucidating more substrates that play key roles in epithelial cell biology and cancer development. Moreover, our study warrants further investigations on the potential of UBA6 downregulation as a biomarker or therapeutic target in a substantial population of clinical breast cancers.

## MATERIALS AND METHODS

### Cell culture and reagents

Human non-transformed mammary epithelial MCF-10A cells were obtained from American Tissue Culture Collection (ATCC). All cells were cultured under standard conditions recommended by ATCC. Horse serum was obtained from HyClone/Thermo Fisher Scientific (Logan, UT), and media, antibiotics and other chemicals were purchased from Corning Cellgro (Manassas, VA) and GiBCO/Invitrogen (Carlsbad, CA).

### Preparation of normal and minimal medium for MCF10A cells

Normal MCF10A culture medium contains DMEM/F12 medium supplemented with 5% horse serum, human EGF (20 ng/mL), insulin (10 μg/mL), cholera toxin (100 ng/mL) and hydrocortisone (0.5 μg/mL). We did pilot experiment to test the effects of withdrawal of the supplemented components in the medium on the formation of “island”-like cell clusters and growth arrest. We cultured MCF10A cells in DMEM/F12 medium with single or combinations of the depletion of phenol red, horse serum, EGF, insulin, cholera toxin and hydrocortisone, and checked the cluster formation and cell growth in MCF10A control cells and sh*UBA6*^Mix^ cells. EGF contributes most to the cluster formation of sh*UBA6*^Mix^ cells, followed by insulin; cholera toxin and hydrocortisone slightly contribute to the cluster formation; phenol red had no visible effect. Depletion of EGF and insulin resulted in efficient growth arrest, whereas simultaneous depletion of EGF, insulin and horse serum rendered cells very sensitive to cell death. In this study, DMEM/F12 medium supplemented with 5% horse serum was used as the minimal medium for most profound effects on growth arrest with minimum cell death in MCF10A control cultures and on the cluster formation in sh*UBA6*^Mix^ cultures.

### Lentiviral overexpression or silencing of UBA6 and UBA1

Lentivirus packaging and transduction of lentiviral vectors for Flag-UBA6, as well as lentiviral silencing of *UBA6* and *UBA1* were conducted as described previously [11].

### Immunoblotting

Preparation of mammalian cell lysates, SDS-PAGE, Western-blot analysis and ECL luminescent detection (GE Healthcare, Little Chalfont, Buckinghamshire, UK) of protein bands were performed as previously described [11]. Anti-Ezrin antibody (#sc-58758, Santa Cruz Biotechnology Dallas, TX), anti-UBA6 mouse monoclonal antibody (#H00055236-M08, Abnova, Walnut, CA), anti-UBA6 rabbit polyclonal antibody (#AP16886b-ev, Abgent), anti-UBA1 antibody (#AP14555a-ev, Abgent, San Diego, CA), anti-α-Tubulin antibody (#T6199, Sigma-Aldrich), anti-Cdk1 antibody (#14341A, BD Pharmingen, Franklin Lakes, NJ), anti-Cdk2 antibody (#sc-163, Santa Cruz Biotechnology), anti-Cdk4 antibody (#sc-260, Santa Cruz Biotechnology), anti-Cdk6 antibody (#sc-177, Santa Cruz Biotechnology), anti-Cyclin A antibody (#Rb0070, Neomarkers, Fremont, California), anti-Cyclin B1 antibody (#RB-008-P0, Neomarkers), anti-Cyclin D1 antibody (#92G2, Santa Cruz Biotechnology), anti-Cyclin E antibody (#sc48, Santa Cruz Biotechnology), anti-CDC25A antibody (#sc7389, Santa Cruz Biotechnology), anti-CDC25C antibody (#MS75p1, Neomarkers), anti-Rb antibody (#sc7905, Santa Cruz Biotechnology), anti-phospho-Rb (Ser807/811) antibody (#9308s, CST), anti-CDC42 antibody (#sc87, Santa Cruz Biotechnology), anti-E-cadherin antibody (#sc-7870, Santa Cruz Biotechnology; #3195, Cell Signaling Technology), γ-catenin (#2309, Cell Signaling Technology), anti-Fibronectin antibody (#MAB 88904, Chemicon/EMD Millipore; #RB-077-A1, Neomarkers), anti-Occludin antibody (#sc-5562, Santa Cruz Biotechnology), anti-N-cadherin antibody (#610920, BD), anti-vimentin antibody (#sc-32322, Santa Cruz Biotechnology; #MS-129-P, NEOMarkers) and anti-Ki67 antibody (#NCL-Ki67p, Leica Biosystems Inc., Buffalo Grove, IL) were used as primary antibodies for immunoblotting or immunofluorescence microscopy.

### Co-immunoprecipitation

The expression vector for HA-tagged UB, pCMV-6His-HA-Ubiquitin, was obtained from Dr. Antonio Iavarone, Columbia University, as a kind gift. The expression vector for CDC42, pCDNAIIIB cdc42 WT (61635), was purchased from Addgene. Co-transfection of the plasmids was conducted using the Lipofectamine® 2000 reagent from Invitrogen (11668-019, Carlsbad, CA), according to the manufacturer’s protocol. For CDC42 immunoprecipitation, cells were treated with 10 μM MG132 (American Peptide, Sunnyvale, CA) for 90 min at 72 h post-transfection, and lysed by sonication in a previously described immunoprecipitation buffer [11]. Lysates with 1mg proteins were incubated with 1 μg antibody overnight at 4°C, followed by incubation with protein G-agarose for 1h at 4°C. Half of the immunoprecipitates were loaded onto an SDS/PAGE gel. The gel was transferred onto a nitrocellulose membrane, and then submerged into boiling water for 10 min for better reactivity of polyubiquitinated proteins with the UB antibody.

### Immunofluorescence microscopy

MCF-10A cells were cultured in 3-D using Matrigel, as previously described [11, 44]. Cell cultures in chamber slides were fixed with 4% paraformaldehyde, followed by staining for immunofluorescence using anti-ezrin antibody (3C12) from Santa Cruz Biotechnology (Santa Cruz, CA), Alexa 488-conjugated anti-mouse IgG and Hoechst 33342 from Life Technologies, Grand Island, NY.

For immunofluorescence using antibodies against the EMT markers and Ki67, MCF10A cells were densely plated on poly-lysine coated coverslips. After 14-day culture in minimal medium, cells were fixed and incubated with relevant primary antibodies and secondary fluorescein-conjugated horse anti-mouse IgG antibody (#FI-2000, Vector Laboratories, Burlingame, CA) or Texas Red^®^-conjugated goat anti-rabbit IgG antibody (#TI-1000, Vector Laboratories). The slides were mounted with Vectashield Mounting Medium plus DAPI (#H-1200, Vector Labs)

Samples were then photographed using Nikon C2 Confocal Microscope or Zeiss Axiovert 200M Microscope. For quantification of the size of acini or “islands”, the images in10x magnification were acquired by TissueFAXS 200 (Tissuegnostics, Vienna, Austria) or by Zeiss Axiovert 200M, followed by analysis using the ImageJ software.

### Bromodeoxyuridine (BrdU) incorporation

Densely cultured MCF10A cells were labeled with 20μM BrdU for 45-60 min. Cells were then fixed and permeabilized, and DNA was denatured by acid treatment, followed by neutralization and rinsing with PBS as described previously [45]. Incorporated BrdU was detected using fluorescein-conjugated anti-BrdU antibody (#51-33284X, Pharmingen, San Diego, CA). Samples were photographed using Zeiss Axiovert 200M.

### Re-culture of 3D acini cells in 2D culture

Three-dimensional spheres or gigantic cell aggregates were isolated from the Matrigel by incubating in Corning cell recovery solution (#354253, Corning, NY) at 4°C for 2 h to dissociate the Matrigel, followed by washing with PBS and treatment with Trypsin (0.05%)/EDTA (0.53 mM) (Corning) to disperse the cells. Cells in suspension were then incubated on plastic culture dishes with normal MCF10A culture medium.

### Analysis of TGF-beta induced EMT

MCF10A control cells or sh*UBA6*^Mix^ cells (3.75×10^5^) were plated on 60-mm culture dishes. EMT was induced by adding 1 ng/mL TGF-β to normal or minimal medium. Cells were split to the starting density every 3 days with fresh addition of TGF-β. Cells were collected at various time points for immunoblotting of the EMT markers.

### Immunohistochemistry on human normal breast and invasive cancer TMAs

TMA blocks were constructed by using the semi-automatic Veridiam Tissue Microarrayer VTA-100 (Veridiam El Cajon, CA USA). The hematoxylin & eosin staining of each donor block was analyzed by pathologists for scribing the area of interest, and each circled area indicated the location in the block from which TMA cores was sampled. A TMA map was created to identify each core’s location in the array. The diameter of each core was adjusted for 2 mm and the donor and the recipient heights were set. Once the area of interest in the block was identified, each area was punched by pressing down slightly on the donor needle holder and transferred into the recipient block. Once the TMA was created, it was subjected to immunohistochemical staining. Tissues from human normal breast and invasive cancer TMA blocks were sectioned at 4 microns and placed on charged slides. Slides were air-dried and placed in a 58–60°C oven for overnight for adhesion of tissue sections, loaded on the Bond Max Microsystems 3.4 Leica instrument (Vision BioSystems Inc. Norwell MA, USA). Samples were then deparaffinized by Bond Dewax solution (AR9222) at 72°C, treated with 100% alcohol and washed by the washing solution (AR9590) for 3 times. After addition of epitope retrieval solution (ER low pH) for 20 minutes at 100°C, samples were then cooled down to ambient temperature for 12 minutes followed by excessive washings and treated with 3% H_2_O_2_ for 5 minutes. UBA6 antibody (1:2000, Cat# H00055236-M08; FLJ10808) (Abnova via Novus Biologicals, LLC, Littleton CO, USA) was applied and the samples were incubated for 15 minutes. Post primary penetration enhancer reagent was added for 8 minutes to the samples followed by addition of Diaminobenzidine (DAB) reagent for 10 minutes. For the counter stain, samples were exposed Hematoxylin for 12 minutes. All slides were rehydrated through alcohol and xylene, mounted and coverslipped (CV5030 Glass Coverslipper, Leica Biosystems Inc., Buffalo Grove, IL, USA). A negative control to each TMA was prepared by using normal mouse IgG instead of the primary antibody. To comparatively examine decreased expression of UBA6 in invasive carcinomas, percentages of UBA6-positive cells in carcinoma regions were scored as follows: 0 (UBA6 cells in carcinoma tissues: 0-5%), 1+ (6%-25%), 2+ (25%-50%), 3+ (>50%). Samples with the scores 0 and 1+ were categorized as the UBA6-low group, whereas those with the scores 2+ and 3+ were grouped as the UBA6-high group. Data were statistically analyzed using Fisher’s exact test and the Wilcoxon rank sum test.

### Data and materials availability

The cell lines generated in this study are available through materials transfer agreements.

## SUPPLEMENTARY MATERIALS FIGURES


