# Supplementary material for: The non-canonical ubiquitin activating enzyme UBA6 suppresses epithelial-mesenchymal transition of mammary epithelial cells
Source: Oncotarget. 2017 Sep 15;8(50):87480–93. doi: 10.18632/oncotarget.20900 (PMC5675648; doi:10.18632/oncotarget.20900)
Supplement: Supplementary file 1 [file oncotarget-08-87480-s001.pdf]

The non-canonical ubiquitin activating enzyme UBA6 suppresses epithelial-mesenchymal transition of mammary epithelial cells

SUPPLEMENTARY MATERIALS

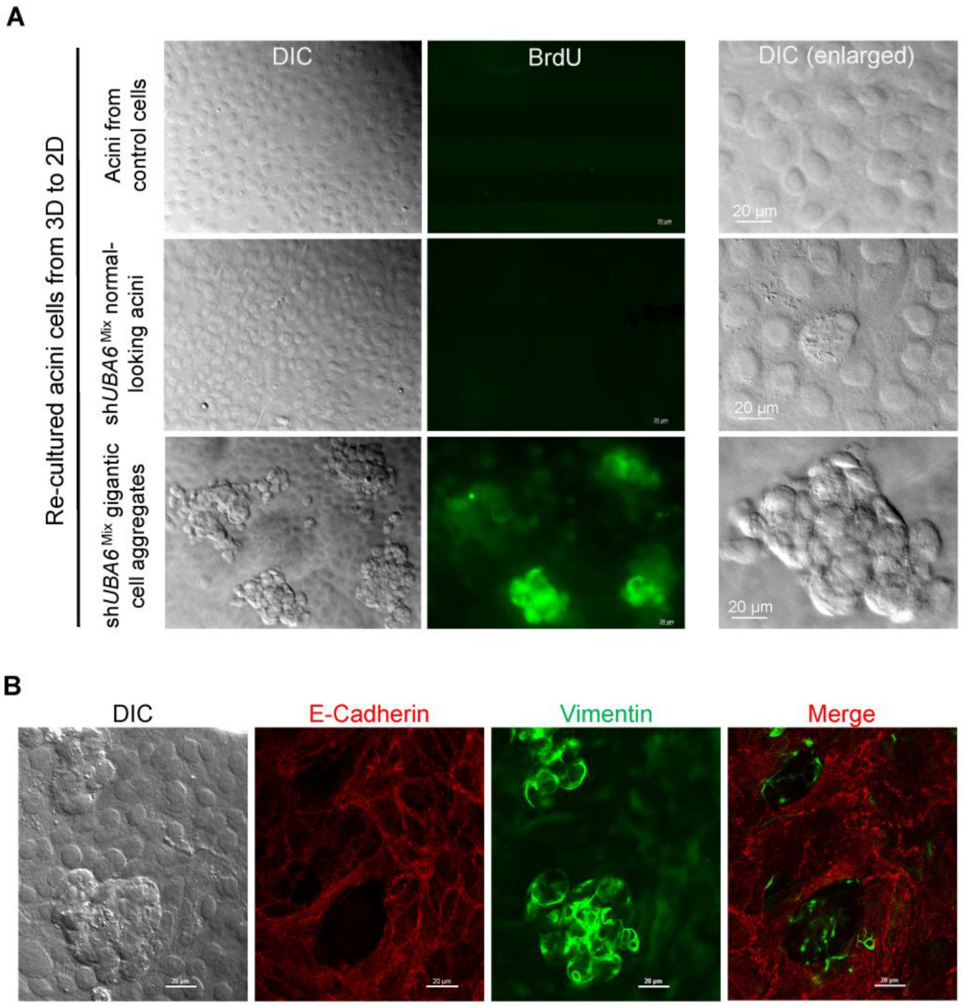

Supplementary Figure 1: The MCF10A cells from 3D gigantic cell aggregates form "island"-like cell clusters and undergo EMT when re-cultured as monolayer in normal medium. **(A)** Representative fluorescence microscope pictures show the formation of "island"-like cell clusters and the enhanced BrdU incorporation in 2D culture only in sh*UBA6* ^{Mix} cells that are re-cultured from 3D gigantic cells aggregates, but not in sh*UBA6* ^{Mix} cells re-cultured from normal-looking acini or in control cells. **(B)** Representative pictures of Immunofluorescence microscopy for EMT markers E-cadherin and Vimentin in cells within the "islands".

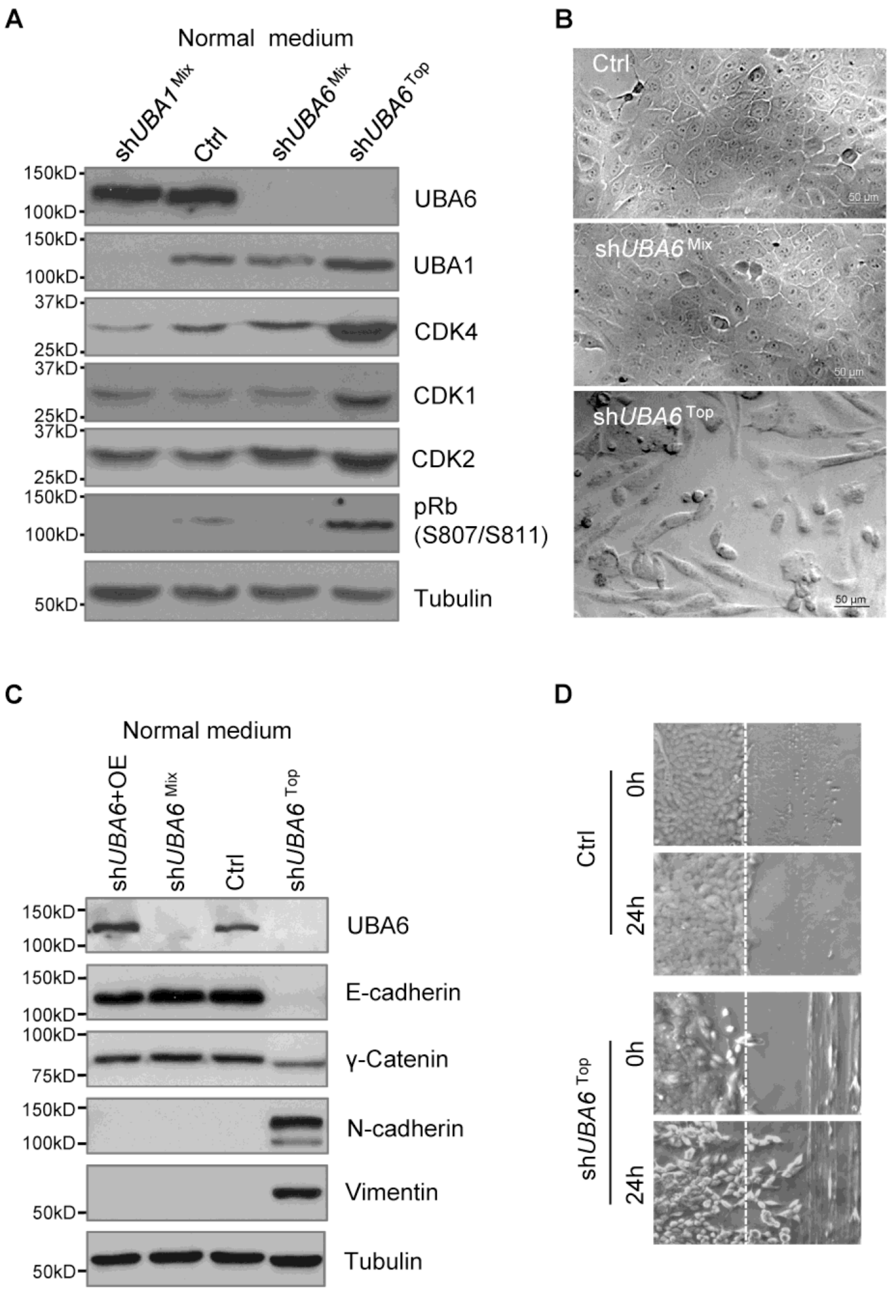

Supplementary Figure 2: UBA6 depletion leads to cell cycle deregulation and EMT in sh*UBA6* Top cells in normal medium. (**A**) Expression of cell cycle-regulatory proteins in sh*UBA6*Top cells. (**B**) Spindle-like morphology in sh*UBA6* Top MCF10A cells (10x magnification). (**C**) EMT markers in sh*UBA6*Top cells. **(D)** sh*UBA6*Top cells show more profound motility than parental MCF-10A cells, in a wound-healing assay in culture. To minimize effects of proliferation, cells were incubated in DMEM/F12 medium supplemented only with 0.25% horse serum for 24 hours in prior to scratching.

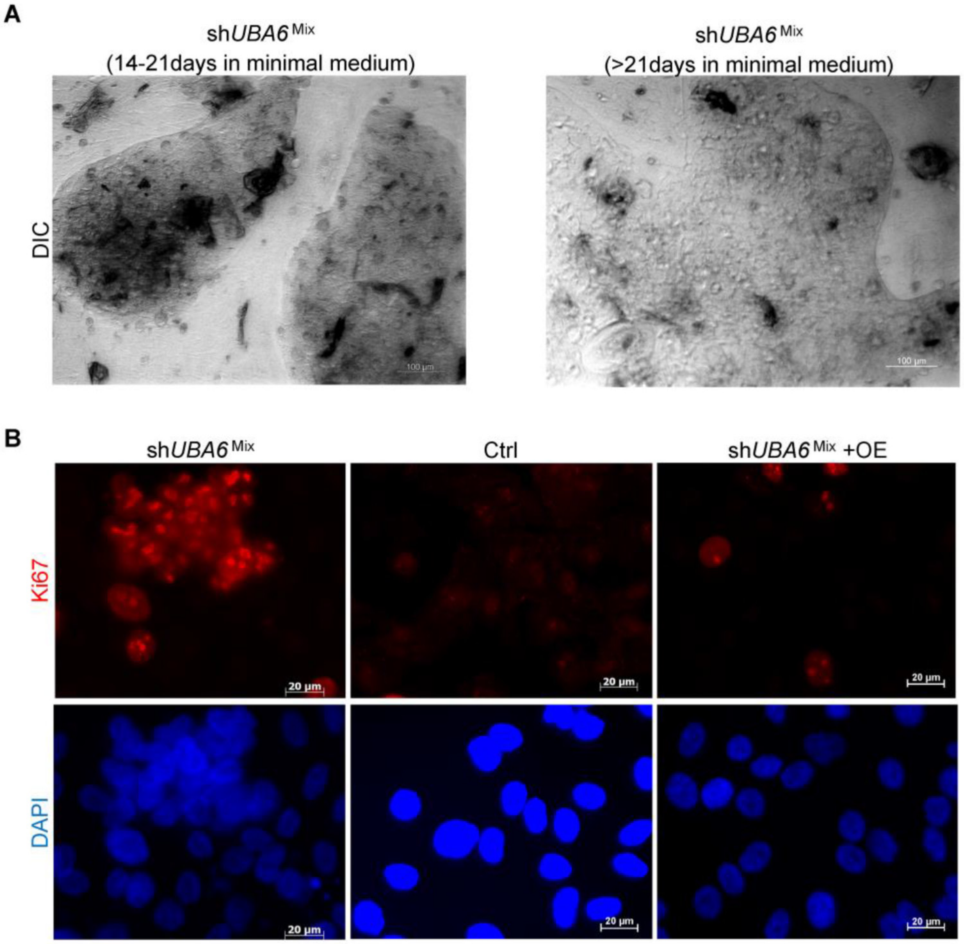

Supplementary Figure 3: UBA6 depletion results in the formation of "island"-like cell clusters that are actively proliferating. (**A**) Representative pictures showing that the "islands" keep expanding their boundaries during prolonged culture in minimal medium in confluent sh*UBA6* cells. (**B**) The MCF10A control cells, sh*UBA6*^{Mix} cells and sh*UBA6*^{Mix} +OE cells were densely plated on poly-lysine coated coverslips and cultured in minimal medium for 14 days, followed by immunofluorescence detection of proliferation marker Ki67. Slides were then photographed using Zeiss Axiovert 200M Fluorescence/Live cell Imaging Microscope (10x magnification).

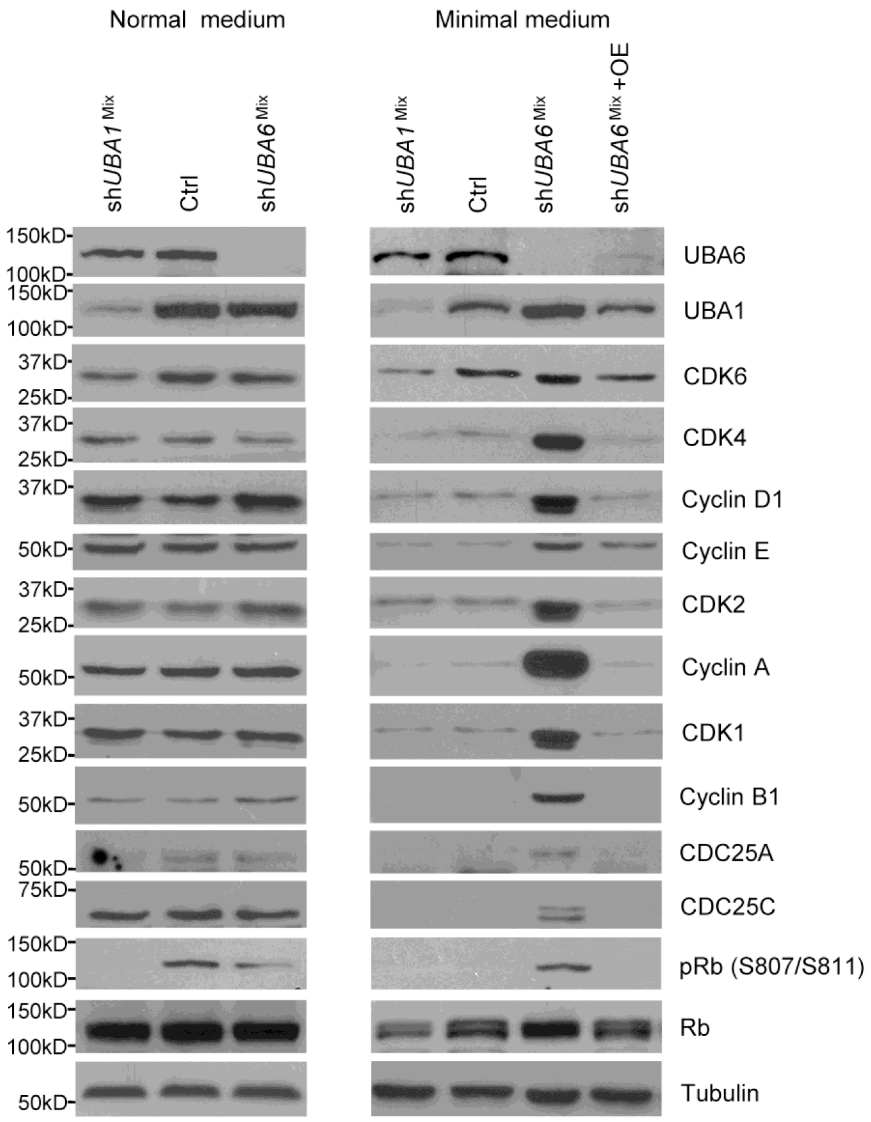

Supplementary Figure 4: UBA6 depletion leads to upregulation of cell cycle regulators in sh*UBA6* ^{Mix} cells in minimal medium. The MCF10A control cells, sh*UBA1* ^{Mix} cells, sh*UBA6*^{Mix} cells and sh*UBA6*^{Mix} +OE cells were cultured in normal medium or in minimal medium for 14 days, followed by immunoblotting for the indicated cell cycle regulators.

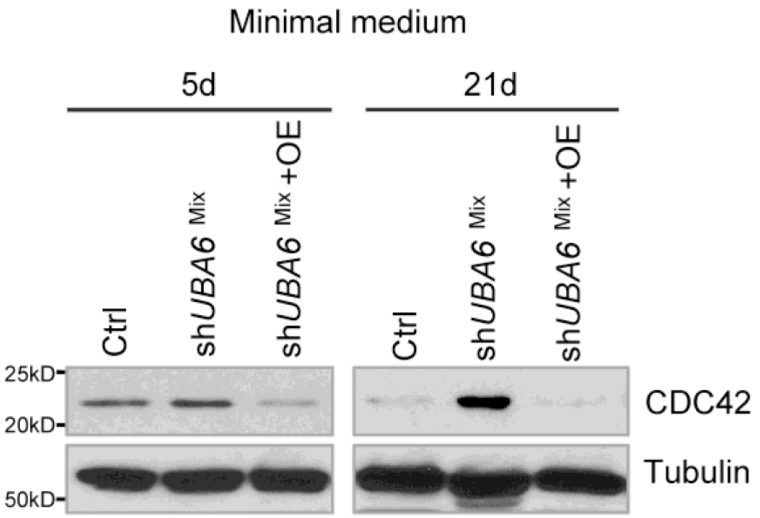

Supplementary Figure 5: CDC42 protein levels are upregulated in sh*UBA6* ^{Mix} cells cultured in minimal medium. The MCF10A control cells, sh*UBA6* ^{Mix} cells and sh*UBA6* ^{Mix} +OE were cultured in minimal medium for 5 or 21 days, followed by immunoblotting for CDC42.

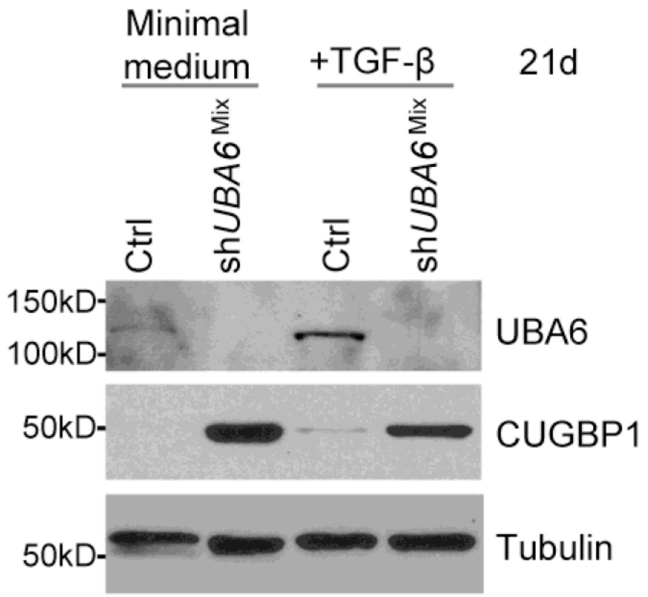

Supplementary Figure 6: Elevated levels of CUGBP1 protein in sh*UBA6* ^{Mix} cells after growth factor deprivation or TGF-β treatment. The MCF10A control cells and sh*UBA6* ^{Mix} cells were cultured in minimal medium or in normal medium plus 1 ng/mL TGF-β for 21 days. The cells were then collected for detection of CUGBP1 by immunoblotting.
